# Beyond candidiasis: should infections caused by *Candidozyma auris* be renamed?

**DOI:** 10.1128/jcm.01530-25

**Published:** 2025-12-23

**Authors:** Yağmur Ekenoğlu Merdan, Kaan Zıkşahna

**Affiliations:** 1 Faculty of Medicine, Department of Medical Microbiology, Biruni University 420479, Istanbul, Türkiye; 2 Biruni Research Center (B@MER) 420479, Istanbul, Türkiye; 3 Department of Bioengineering, Faculty of Chemical and Metallurgical Engineering, Yildiz Technical University 406501 , Istanbul, Türkiye; 4 Faculty of Medicine, Department of Basic Medical Sciences, Biruni University, Istanbul, Türkiye; Vanderbilt University Medical Center, Nashville, Tennessee, USA

**Keywords:** *Candidozyma auris*, emerging fungal infections, fungal nomenclature, taxonomic reclassification

## LETTER TO EDITOR

In their recent article, de Hoog et al. (1) proposed a standardized framework for naming fungal pathogens and diseases. In light of this important discussion, we wish to comment on the nomenclature of *Candidozyma auris* infections, a recently reclassified species.

The emerging fungal pathogen *C. auris* is leading to serious outbreaks of invasive disease in healthcare facilities globally. *C*. *auris* has become a major concern in critical care settings, particularly among immunocompromised patients (2). The National Center for Biotechnology Information (NCBI) Taxonomy page outlines the current taxonomic status of *C. auris* (3). The species was reclassified, officially reported to be in the genus *Candidozyma* by Liu et al. in 2024, and placed within the family Metschnikowiaceae (4).

Candidiasis is an infection caused by *Candida* species. Recent taxonomical changes due to the introduction of molecular techniques for identification have led to the renaming of *C. auris* into separate genera (4). The reclassification of *Candidozyma auris* has important implications for clinical terminology. This yeast exhibits unique characteristics that differ substantially from other members of the former *Candida* genus, including inability to proliferate in the anaerobic environment of the human gut, high transmissibility, multidrug resistance, persistence on hospital surfaces, and the capacity to cause large-scale outbreaks among critically ill or immunocompromised patients (5–8). The use of the term candidiasis, traditionally used for infections caused by *Candida* species, is thought to have become increasingly ambiguous following the reclassification of *C. auris*. This raises important questions: should a distinct term be used to describe infections caused specifically by *C. auris*? Will the term *candidiasis* remain valid for this species despite its removal from the genus *Candida*?

Two contrasting perspectives have been proposed regarding the naming of fungal diseases. Odds et al. (9) argued that a change in the taxonomic name of a fungus should not entail a change in the corresponding disease name, as this could misleadingly suggest a change in the disease itself and hinder clinical communication. He therefore recommended using the new organism name together with the established disease term, which would be both scientifically and clinically appropriate. In contrast, Denning (10) and de Hoog et al. (1) emphasized that taxonomic reclassification of fungi often leads to inconsistencies between the names of diseases and their etiologic agents, creating challenges for diagnosis, epidemiology, and public health surveillance. For reclassified species such as *C. auris*, continued use of the term *candidiasis* may no longer reflect its distinct biology, suggesting that a terminology update may be needed.

Given these differing perspectives, it becomes important to re-evaluate whether long-used terminology adequately represents reclassified and clinically differentiated fungi such as *C. auris*. We propose that terms such as “candidozosis” or “candidozymiasis” be evaluated for adoption in scientific and clinical contexts. Establishing a consistent terminology would not only align with current taxonomy but also improve clarity in epidemiological surveillance, clinical reporting, and antifungal stewardship efforts.

The reclassification of *C. auris* highlights the tension between taxonomic accuracy and clinical terminology. While maintaining established disease names aids clarity, the distinct biology of *C. auris* warrants reconsideration. A unified, evidence-based approach is needed to ensure consistency in taxonomy and clinical communication.
